# Towards atomically-thin regime in bulk 4H-NbSe_2_ with interlayer disorder

**DOI:** 10.1038/s41699-025-00659-w

**Published:** 2026-01-06

**Authors:** Edoardo Martino, Alla Arakcheeva, Helmuth Berger, Yuri Prots, Markus König, László Forró, Konstantin Semeniuk

**Affiliations:** 1 École Polytechnique Fédérale de Lausanne (EPFL), Institute of Physics, Lausanne, Switzerland; 2 Max Planck Institute for Chemical Physics of Solids, Dresden, Germany; 3 Stavropoulos Center for Complex Quantum Matter, University of Notre Dame, Notre Dame, IN USA

**Keywords:** Electronic properties and materials, Superconducting properties and materials

## Abstract

Polytypism in transition metal dichalcogenides (TMDs) introduces an additional degree of freedom for tailoring the electronic properties of layered van der Waals materials. Polytypes with larger unit cells, spanning four or six layers, can be viewed as natural homostructures, since their atomic composition remains identical across the layers. The resultant crystalline environments can potentially give rise to exotic electronic states, earning these materials recent attention. In this study, we examine structural and charge transport properties of metallic and superconducting 4H_a_-NbSe_2_. We find that the compound has a highly disordered stacking of layers, which impedes interlayer coherence, as demonstrated by detailed out-of-plane resistivity measurements, and effectively tunes the bulk system towards an atomically thin limit. The disordered structure largely accounts for the enhanced resistivity anisotropy and superconducting upper critical field, when compared to 2H_a_-NbSe_2_. This phenomenon can be exploited to promote quasi-two-dimensional physics in bulk crystals, and our study also underscores the importance of thorough structural characterization when investigating large-unit-cell polytypes of TMDs.

## Introduction

The ease of exfoliating layered van der Waals materials into atomically thin crystalline sheets makes them promising building blocks for designing functional metamaterials^1^. Weak interlayer coupling enables the creation of stable structures with diverse stacking orders. In transition metal dichalcogenides (TMDs), this property manifests naturally as a pronounced polytypism: by slightly altering the crystal growth conditions, one can synthesize materials with the same chemical formula but different metal-chalcogen coordination and different arrangements of layers along the normal *c*-axis^2,3^. With unit cells reaching four or even six layers, some aspects of layered metamaterials can be realized in a conventionally synthesized crystal. A recent example of this is 4H_b_-TaS_2_, which has been proposed to host an exotic superconducting state^4,5^.

The fact that various polytypes of TMDs have comparable enthalpies of formation also makes it more probable for a synthesized crystal to simultaneously host multiple competing stacking orders, leading to frequent stacking faults^6^, which can considerably affect electronic properties^7^. Polytypes with larger unit cells typically form at higher temperatures and require rapid quenching at the end of the growth to avoid transitions into more stable polytypes upon cooling^8^. Nonetheless, when different structures are particularly close in energy, a significant presence of structural defects in the final crystal can be anticipated. This issue is especially relevant for the 4H class of polytypes, with at least five different substructures proposed in the literature^2^. Despite the growing attention to these structurally complex systems, the extent of static disorder in 4H polytypes of TMDs and its effect on electronic properties is scarcely documented.

In this work, we correlate the structural and electronic properties of 4H_a_-NbSe_2_. Crystallographic characterization shows that while individual layers are structurally identical to those of 2H-NbSe_2_, the crystal is heavily disordered along the *c* axis, which is also confirmed by the resistivity anisotropy data. Frequent planar defects effectively reduce the dimensionality of the system, tuning the charge density wave (CDW) instability and superconductivity in the compound towards the regime realized in atomically thin 2H_a_-NbSe_2_^9^.

## Results

### X-ray

**Fig. 1:**
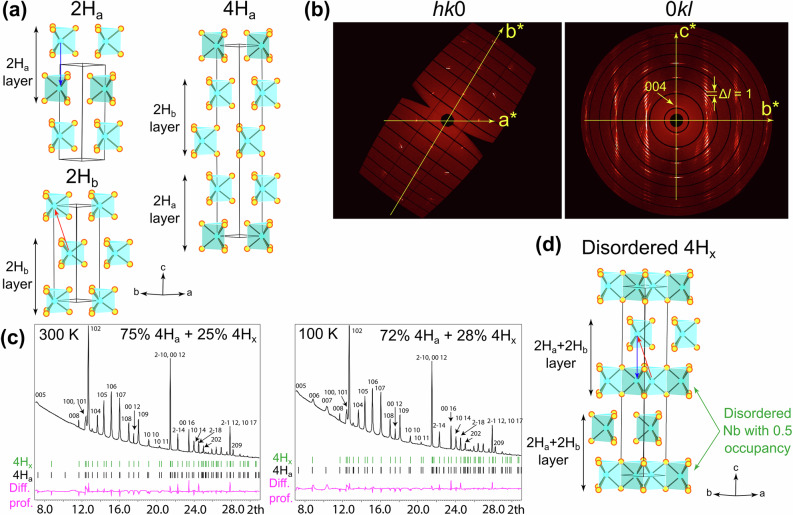
Structural information on 4H_a_-NbSe_2_crystals. **a** Crystalline structure of 2H_a_, 2H_b_, and 4H_a_ polytypes of layered transition metal dichalcogenides. In the case of NbSe_2_, the Nb and Se atoms correspond to the cyan and yellow circles, respectively. **b** Single crystal X-ray diffraction patterns of 4H_a_-NbSe_2_ in the *hk*0 and 0*kl* planes of the reciprocal space. **c** Powder X-ray diffraction profiles at 300 K and 100 K with the Rietveld refinement. **d** Crystalline structure of a disordered phase 4H_x_-NbSe_2_ used for fitting the powder X-ray diffraction profiles. The structures were visualized with the help of *Diamond* (Version 3) software by CRYSTAL IMPACT. *CrysAlisPro*1.171.40.68a by Rigaku Oxford Diffraction was used for the reciprocal space reconstructions. The powder diffraction plots were produced using JANA 2006 software^33^, which was also used for the refinement of the crystallographic data.

Out of all the proposed polytypes of layered NbSe_2_, only two have been synthesized in bulk and stoichiometric forms and had their electronic properties studied at different temperatures: 2H-NbSe_2_^10^ and 4H-NbSe_2_^11^. These two compounds have two- and four-layer unit cells, respectively, and while these designations are widely used throughout the literature, here, we adopt a more precise notation. For 2H-NbSe_2_, the exact polytype is 2H_a_, corresponding to the $P{6}_{3}/{mmc}$ symmetry group^12^. In 2H_a_-NbSe_2_, the metal atoms are aligned along the *c* axis and the orientation of the trigonal prismatic NbS_6_ clusters alternates between neighboring layers, as illustrated in Fig. 1a, where we also show the 2H_b_ structure. While various layer-stacking configurations have been previously proposed for 4H-NbSe_2_^2^, recent works point to 4H_a_-NbSe_2_ ($P\bar{6}m2$ symmetry group) being the precise polytype^13^. As shown in Fig. 1a, the 4H_a_ structure can be viewed as an alternating sequence of 2H_a_ and 2H_b_ bilayers.

We conducted an X-ray diffraction study of 4H_a_-NbSe_2_ in nominally single-crystal and powder forms. The single crystal diffraction patterns in the *hk*0 and 0*kl* planes are shown in Fig. 1b. For the *hk*0 plane, we observed a triangular lattice of reflections with numerous satellites, indicating that the sample is not a single crystal, but rather consists of multiple blocks sharing the *c*-axis direction, while their *a* and *b* axes are misaligned. In the 0*kl* plane, while the 4-layer periodicity is present, the pronounced circular smearing of the pattern indicates a particularly strong disorder along the *c*-axis.

Further structural information was obtained via the powder X-ray diffraction (Fig. 1c). Broad 00*l* reflections with odd *l* violate the $P{6}_{3}/{mmc}$ symmetry group but not the $P\bar{6}m2$ one. Their emergence at a lower temperature of 100 K suggests a strengthening of the long-range order upon cooling. The overall pattern is consistent with the 4H_a_-NbSe_2_ structure, making it possible to index every prominent peak. Our measured spectra are in reasonably good agreement with another crystallographic study of the compound^13^, which mentions the broadening of the 10*l* reflections as an indication of stacking faults.

With the help of Rietveld refinement, we obtained more detailed and quantitative information regarding the statistically most likely type of defect in our crystal. As indicated in Fig. 1c, the difference profiles at 300 K and 100 K were minimized by fitting a mix of two crystalline phases: the pristine 4H_a_-NbSe_2_ as well as the disordered 4H_x_-NbSe_2_. The latter structure is visualized in Fig. 1d, and its unit cell can be schematically broken down into bilayers, each of which is a superposition of 2H_a_ and 2H_b_ structures with a 50% occupancy of certain Nb sites. The relative content of the 4H_a_ and 4H_b_ phases is 75:25 at 300 K and 72:28 at 100 K. For more detailed results of the Rietveld refinement analysis, refer to the Supplementary Information (Supplementary Table 1; Supplementary Figs. 1, 2 and Supplementary Files 1, 2).

### Resistivity anisotropy

**Fig. 2:**
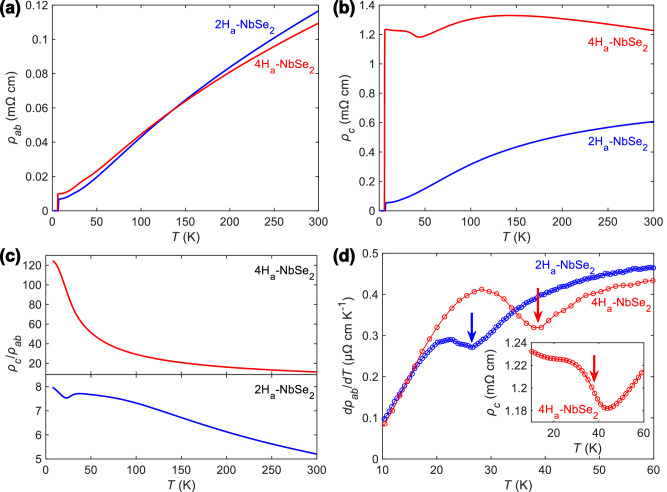
Comparison of charge transport in 2H_a_-NbSe_2_ and 4H_a_-NbSe_2_. In-plane (**a**) and out-of-plane (**b**) resistivities (*ρ*_*ab*_ and *ρ*_*c*_, respectively) of 2H_a_-NbSe_2_ and 4H_a_-NbSe_2_ as functions of temperature (*T*). **c** Temperature dependences of resistivity anisotropies of the two compounds. **d** Signatures of the charge density wave (CDW) transitionsin 2H_a_-NbSe_2_ and 4H_a_-NbSe_2_ seen in the derivative of *ρ*_*ab*_ with respect to *T*. Inset: *ρ*_*c*_ of 4H_a_-NbSe_2_ across the CDW transition.

The abundance of static disorder along the *c-*axis in the 4H_a_-NbSe_2_ crystals is bound to result in a strongly enhanced scattering of charge carriers, affecting the out-of-plane electrical resistivity of the material. We measured and compared the temperature ($T$) dependences of resistivities of 2H_a_-NbSe_2_ and 4H_a_-NbSe_2_ crystals, examining the influence of the defects on the interlayer conduction. Similarly to previous studies of resistivity anisotropy in TMDs^7,14^, we used samples of well-defined geometry, produced with focused-ion-beam (FIB) microscopy^15^. We found that in-plane resistivity, ${\rho }_{{ab}}(T)$, is very similar in the two polytypes both in terms of their absolute values and temperature dependences (Fig. 2a). Such a similarity is expected based on the identical structures of the individual layers of the compounds. The studied 2H_a_-NbSe_2_ sample had a slightly higher residual resistivity ratio of 16.6, compared to 12.4 for the 4H_a_-NbSe_2_ one (the ratio is taken between resistances at 300 K and 7 K). Assuming the partial occupancy of certain Nb sites in the real 4H_a_-NbSe_2_ crystals, suggested by the crystallographic analysis, scattering of charge carriers in the material is expected to be enhanced compared to the 2H_a_ polytype even for the in-plane transport. The CDW transition, which occurs at a temperature of 33 K in 2H_a_-NbSe_2_, is shifted to 42 K in 4H_a_-NbSe_2_ (Fig. 2d). This shift has previously been attributed to an enhancement of electron-phonon coupling^13^, which could be driven by a reduction of dimensionality^16,17^.

The high density of stacking faults manifests as a strongly enhanced and weakly temperature-dependent out-of-plane resistivity, causing a dramatic difference in ${\rho }_{c}(T)$ between the two polytypes (Fig. 2b). For 2H_a_-NbSe_2_, ${\rho }_{c}(T)$ is clearly metallic, with the anisotropy ${\rho }_{c}/{\rho }_{{ab}}$ increasing from approximately 5–8 between 300 K and 7 K (Fig. 2c). For 4H_a_-NbSe_2_, ${\rho }_{c}(T)$ is dominated by a residual contribution of about 1.2 mΩ cm, and varies with temperature in a relatively narrow interval of approximately 0.2 mΩ cm. As a result, the anisotropy increases from 10 to more than 120 upon cooling. These data clearly align with the abundance of planar defects in 4H_a_-NbSe_2_, as indicated by the X-ray study.

Interestingly, ${\rho }_{c}(T)$ of 4H_a_-NbSe_2_ shows a significantly stronger response to the CDW instability than that of 2H_a_-NbSe_2_. The CDW transition is known to cause the *c*-axis position of Se atoms to become modulated throughout a layer^18^, but if these atoms in two neighboring layers misaligned along the *c*-axis (which is the case for the ideal 2H_a_ and 4H_a_ structures), they mostly avoid each other as the distortion takes place. Consequently, 2H_a_-NbSe_2_ does not exhibit a strong change in the *c*-axis lattice constant across the transition^19^, which is in line with the lack of any visible feature in ${\rho }_{c}(T)$ at 33 K. The anomaly in ${\rho }_{c}(T)$ of 4H_a_-NbSe_2_ at 42 K could therefore originate from processes happening at the stacking defects, where the interlayer conduction is bottlenecked.

### Superconductivity

**Fig. 3:**
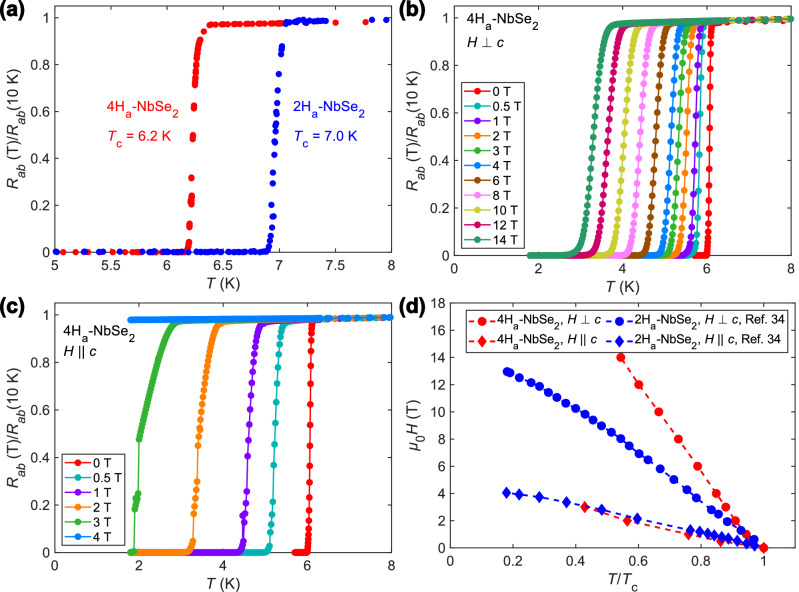
Superconducting critical temperatures and fields in 4H_a_-NbSe_2_. **a** In-plane resistances of 2H_a_-NbSe_2_ and 4H_a_-NbSe_2_ as functions of temperature (*T*), normalized to the 10 K values, showing the superconducting transitions. Resistive superconducting transition of 4H_a_-NbSe_2_ for different in-plane (**b**) and out-of-plane (**c**) magnetic fields. **d** In-plane and out-of-plane superconducting upper critical field curves of 4H_a_-NbSe_2_ and 2H_a_-NbSe_2_ according to the present data and an earlier study^34^, respectively (*H*–magnetic field, *μ*_0_–vacuum permeability). The temperature axis is scaled by the zero-field value of the critical temperature (*T*_c_) of each compound.

In light of the new structural information on 4H_a_-NbSe_2_, we examined the superconducting properties of the material. The superconductivity occurs in both 4H_a_ and 2H_a_ polytypes, and the critical temperatures (${T}_{\mathrm{c}}$) found in our samples were 6.2 K and 7.0 K, respectively (Fig. 3a).

When a magnetic field is applied along the *c*-axis, 4H_a_-NbSe_2_ shows effectively the same critical field curve as 2H_a_-NbSe_2_, when plotted against the reduced temperature $T/{T}_{\mathrm{c}}$. Since for the *c*-axis field the orbital motion of electrons occurs within the *ab* plane, it is natural to expect the two polytypes to show similar behaviors. The in-plane critical field of 4H_a_-NbSe_2_ is, however, strongly enhanced compared to 2H_a_-NbSe_2_: for example, at $T/{T}_{\mathrm{c}}$ = 0.6, the superconductivity is destroyed at 7 T in the 2H_a_ polytype, but persists to 12.5 T in the 4H_a_ polytype. Such an enhancement has been observed in previous studies of the compound^11,13^, and was also found to occur in atomically thin 2H_a_-NbSe_2_^9^. In both cases, the same physics can explain the enhanced in-plane critical field. The lack of inversion symmetry at the Nb site locally lifts the spin degeneracy of electronic bands via the Ising spin-orbit coupling and favors the out-of-plane alignment of spins. The Pauli paramagnetic pair-breaking field for *H*⟂*c* is therefore strongly enhanced compared to the BCS weak coupling value. This enhancement, characteristic even for a pristine 4H_a_-NbSe_2_^20^, causes the orbital pair-breaking to be dominant, which is reflected by the critical field curve staying linear over a large interval below ${T}_{\mathrm{c}}$. Reducing sample thickness to a few layers or introducing frequent stacking faults further increases the in-plane critical field by hindering the out-of-plane motion of electrons, thus making the orbital pair-breaking less effective.

## Discussion

We now discuss microscopic effects which can explain how interlayer disorder in a bulk crystal leads to the enhanced two-dimensionality of the electronic properties. The large extent of stacking disorder in 4H_a_-NbSe_2_, expected based on the metastability of the polytype, causes extreme changes to the interlayer coupling compared to the more stable 2H_a_ polytype. The out-of-plane resistivity of 2H_a_-NbSe_2_ displays a coherent metallic transport, also observed in some other TMDs^7,14^. The moderate resistivity anisotropy ${\rho }_{c}/{\rho }_{{ab}}$, not exceeding 10, is unsurprising given the existence of a pancake-shaped Fermi pocket around the Γ point of the Brillouin zone^21^. Using experimental data and taking the *c*-axis scattering rate of 2000 cm^−1^ at 300 K^22^, as well as the out-of-plane Fermi velocity of the order of 10^4 ^ms^−1^ ^23,24^, one can get a rough estimate of the out-of-plane mean free path of less than 1 nm at room temperature. With the *c*-axis lattice constant of 1.25 nm, spanning two layers, the Mott-Ioffe-Regel limit of resistivity is expected to be approached at high temperatures, which is consistent with the visible flattening of the ${\rho }_{c}(T)$ curve upon warming up the sample (Fig. 2b).

In the case of 4H_a_-NbSe_2_, the room-temperature value of ${\rho }_{c}$ is approximately twice higher than for the 2H_a_ polytype. This suggests an even shorter out-of-plane mean free path, with a significant chance for a charge carrier to become scattered at every layer. Such a high density of stacking faults is also consistent by the suppression of the superconducting ${T}_{\mathrm{c}}$ in 4H_a_-NbSe_2_ compared to the 2H_a_ polytype. The critical temperature is known to be reduced in atomically thin 2H_a_-NbSe_2_, and ${T}_{\mathrm{c}}$ of 6.2 K in 4H_a_-NbSe_2_ approximately corresponds to a 4-layer-thick film^9^, which is roughly in line with the density of defects predicted by our X-ray analysis.

While the high density of static defects makes ${\rho }_{c}(T)$ of 4H_a_-NbSe_2_ mostly temperature-independent, one can still see its slope varying in sign in different temperature ranges, which suggests that multiple conduction mechanisms are at play. One can imagine that the coherent interlayer hopping, facilitated by the Nb ${d}_{{z}^{2}}$ orbitals^25^, is locally disrupted by planar defects as well as the partial Nb site occupancy. Since ${\rho }_{c}(T)$ does not follow a divergent form at low temperature, the conduction cannot be described solely by the thermally activated or tunneling transport, so the metallic *c*-axis conduction channel, spanning the entire sample, appears to persist at least in some fraction of the crystal. The disruption of the *c*-axis periodicity may hinder the formation of long-range itinerant Bloch states, resulting in the negative temperature coefficient of resistivity, generally observed in quasicrystals and highly amorphous metals^26,27^.

The case of simultaneous incoherent interlayer and coherent intralayer transport in layered materials has previously been considered in context of normal state resistivity anisotropy of certain cuprate superconductors^28,29^. Assuming ${t}_{c} < h{\tau }_{{ab}}^{-1} < {t}_{{ab}}$ (${t}_{c}$, ${t}_{{ab}}$ – out-of-plane and in-plane hopping parameters, respectively; ${\tau }_{{ab}}^{-1}$ – in-plane scattering rate, $h$ – Planck constant), the *c*-axis conduction can be dominated by the tunneling events which occur as a result of the in-plane scattering of charge carriers. If the tunneling occurs between the sites of the same energy, then ${\rho }_{c} \sim {\left|{t}_{c}\right|}^{2}{\tau }_{{ab}}^{-1}$, meaning that the temperature dependence of ${\rho }_{c}$ mimics the conventional metallic behavior of ${\rho }_{{ab}}$. However, if the initial and final states are separated by $\Delta E$ in energy, or if an intermediate state of a different energy is involved, the tunneling contribution to ${\rho }_{c}$ becomes further scaled by ${e}^{\left(\Delta E/{k}_{\mathrm{B}}T\right)}$ and diverges at low temperatures. The shift of electronic transport from strongly coherent in 2H_a_-NbSe_2_ towards the incoherent regime in 4H_a_-NbSe_2_ may explain the enhanced two-dimensionality of the latter system.

In our investigation of structural and change transport properties of 4H_a_-NbSe_2_, we demonstrated that while individual layers of the compound are effectively the same as in 2H_a_-NbSe_2_, the 4H_a_ polytype has a high density of stacking faults, which severely impact the electronic interlayer coherence. This disorder is a consequence of a higher synthesis temperature, making the notion relevant for a variety of metastable polytypes across numerous TMDs, and calling for a more comprehensive structural characterization of such systems.

As a result of abundant stacking faults, the two-dimensionality of 4H_a_-NbSe_2_ crystals is enhanced, causing the CDW transition and superconductivity in 4H_a_-NbSe_2_ to behave similarly to what is observed in a few-layer-thick 2H_a_-NbSe_2_. We emphasize that the observed effects on the stability of the ordered states cannot be attributed to a static disorder alone, which would typically smear out phase transitions, but rather to the reduced dimensionality as a result of the stacking faults.

Inducing stacking disorder in van der Waals materials while preserving the purity of individual layers could be a route to realizing low-dimensional superconductivity and related phenomena in bulk systems^30,31^, thus bypassing the need for exfoliation and enabling their investigation by a wider range of probes.

## Methods

### Synthesis

4H_a_-NbSe_2_ single crystals were prepared by Chemical Vapour Transport with an excess of selenium in a sealed quartz tube under an inert atmosphere at 900 °C for multiple days.

### X-ray diffraction measurements

The X-ray diffraction data were collected at the BM01 Swiss–Norwegian beamline of the European Synchrotron Radiation Facility (ESRF) in Grenoble. The beam had a wavelength of 0.64109 Å and a 100 × 100 nm size. PILATUS@SNBL detector^32^ was used for signal acquisition and the sample-detector distance was 146 mm.

### Electrical resistivity measurements

Measurements of electrical resistivity were conducted in a Quantum Design PPMS cryostat, using the DC resistivity option. Excitation currents did not exceed 0.2 mA in order to avoid any noticeable Joule heating of the samples. The preparation of samples with the focused ion beam was conducted according to the same protocol as in our earlier study in ref. ^7^. The probed sections of the crystals had widths and thicknesses of a few µm, and lengths close to 10 µm (out-of-plane channels) or 50 µm (in-plane channels). The four-point resistances of the samples were mostly in the 1–10 Ω range.

## Supplementary information

Supplementary information

Supplementary file 1

Supplementary file 2

## Data Availability

Data supporting the results of this paper can be provided by K.S. upon a reasonable request.

## Code availability

Code used for processing the data in this work can be provided by K.S. upon a reasonable request.
