# Supplementary material for: Towards atomically-thin regime in bulk 4H-NbSe2 with interlayer disorder
Source: NPJ 2D Mater Appl. 2026 Jan 6;10(1):23. doi: 10.1038/s41699-025-00659-w (PMC12885948; doi:10.1038/s41699-025-00659-w)
Supplement: Supplementary file 1 — Supplementary information [file 41699_2025_659_MOESM1_ESM.pdf]

Towards atomically-thin regime in bulk 4H-$NbSe_2$ with interlayer disorder

Supplementary Information

Edoardo Martino[1], Alla Arakcheeva[1], Helmuth Berger[1], Yuri Prots[2], Markus König[2], László Forró[3,1], Konstantin Semeniuk[2,1]

1. *École Polytechnique Fédérale de Lausanne (EPFL), Institute of Physics, CH-1015, Lausanne, Switzerland*
2. *Max Planck Institute for Chemical Physics of Solids, 01187 Dresden, Germany*
3. *Stavropoulos Center for Complex Quantum Matter, University of Notre Dame, Notre Dame, 46556, IN, USA*

Supplementary table 1

The table below contains the structural data based on Rietveld refinement.

**Phase**	**T (K)**	**Space group**	**Parameters a, c (Å)**	**Atomic coordinates**	**Characteristics of Rietveld refinement**	**Remarks**	**Reference**
2H(a)	290	$P6_3/mmc$	*a* = 3.446,<br>*c* = 12.554	Nb: 0 0 ¼<br>Se: 1/3 2/3 0.1160		The structure and the simulated powder XRD profile are shown in Supplementary figures 1,2.	Simulation after [S1] CCD collection code 129897
2H(b)	290	$P\text{-}6m2$	*a* = 3.4425,<br>*c* = 12.547	Nb1: 0 0 0<br>Nb2: 1/3 2/3 ½<br>Se1: 1/3 2/3 0.1161<br>Se2: 2/3 1/3 0.3839	--	The structure and the simulated powder XRD profile are shown in Supplementary figures 1,2.	Simulation after [S2] CCD collection code 201319
2H(c)	290	$P6_3/mmc$	*a* = 3.4425,<br>*c* = 12.547	Nb: 1/3 2/3 ¼<br>Se: 2/3 1/3 0.1161	--	The structure and the simulated powder XRD profile are shown in Supplementary figures 1,2.	Simulation
4H(a)	290	$P\text{-}6m2$	*a* = 3.4432(8),<br>*c* = 25.2294(7)	Nb1 (1*a*): 0 0 0<br>Nb2 (1*f*): 2/3 1/3 ½<br>Nb3 (2*g*): 0 0 0.2492(6)<br>Se1 (2*i*): 2/3 1/3 0.0664(7)<br>Se2 (2*h*): 1/3 2/3 0.3210(3)<br>Se3 (2*h*): 1/3 2/3 0.1846(8)<br>Se4 (2*g*): 0 0 4303(1)	R_p = 4.64% ;<br>R_{wp} = 7.42% ;<br>No *R*1 is shown in the publication	Our present work shows much better profile fit for both R_p and R_{wp} even for only a single 4H(a) phase at 100 K.	After [S3]
Two phases:<br>(i) 75(2)% **4H(a)**<br>(ii) 25(2)% **4H(x)**	300	**4H(a)**: $P\text{-}6m2$;<br>**4H(x)**: $P\text{-}62c$	**4H(a)**:<br>*a* = 3.4440(6),<br>*c* = 25.240(9);<br>**4H(x)**:<br>*a* = 3.4443(5),<br>*c* = 25.240(5)	**4H(a)**:<br>Nb1 (1*a*): 0 0 0<br>Nb2 (1*f*): 2/3 1/3 ½<br>Nb3 (2*g*): 0 0 0.247(2)<br>Se1 (2*i*): 2/3 1/3 0.054(3)<br>Se2 (2*h*): 1/3 2/3 0.295(5)	*R*p = 1.30 %;<br>*wR*p = 2.53 %;<br>For **4H(a)** ***R*1** = 8.15%;<br>for **4H(x)** ***R*1** = 11.22%	The structure and the simulated powder XRD profile of the 4H(x) phase are shown in Supplementary figures 1,2. The CIF file of the	Present work

				Se3 (2*h*): 1/3 2/3 0.194(5)<br>Se4 (2*g*): 0 0 435(3);<br>**4H(x)**:<br>Nb1 (2*d*): 1/3 2/3 3/4<br>0.5* Nb2 (4*f*): 1/3 2/3 0.502(2)<br>Se1 (4*e*): 0 0 0.571(3)<br>Se2 (4*f*): 2/3 1/3 0.680(1)		mixed phase is available as Supplementary file 1.<br>(2) 4H(x) reflects disorder in stacking fault between 4H(a).<br>(3) Two the indicated phases essentially improve fit of the observed profile, both *R*p and *wR*p (see the profiles).	
**4H(a)** Single Phase	100	*P*-6*m*2	*a* = 3.4369(3),<br>*c* = 25.15(1)	Nb1 (1*a*): 0 0 0<br>Nb2 (1*f*): 2/3 1/3 ½<br>Nb3 (2*g*): 0 0 0.248(2)<br>Se1 (2*i*): 2/3 1/3 0.066(4)<br>Se2 (2*h*): 1/3 2/3 0.32(2)<br>Se3 (2*h*): 1/3 2/3 0.18(2)<br>Se4 (2*g*): 0 0 431(4)	*R*p = 2.65 %;<br>*wR*p = 6.08 %;<br>*R*1 = 7.19%	The structure and the simulated powder XRD profile are shown in Supplementary figures 1,2.	Present work
Two phases:<br>(i) 72(3)% **4H(a)**<br>(ii) 28(3)% **4H(x)**	100	**4H(a)**: *P*-6*m*2;<br>**4H(x)**: *P*-62*c*	**4H(a)**:<br>*a* = 3.4373(9),<br>*c* = 25.155(16);<br>**4H(x)**:<br>*a* = 3.4378(6),<br>*c* = 25.154(6)	**4H(a)**:<br>Nb1 (1*a*): 0 0 0<br>Nb2 (1*f*): 2/3 1/3 ½<br>Nb3 (2*g*): 0 0 0.248(2)<br>Se1 (2*i*): 2/3 1/3 0.054(4)<br>Se2 (2*h*): 1/3 2/3 0.291(6<br>Se3 (2*h*): 1/3 2/3 0.196(7)<br>Se4(2*g*): 0 0 435(4);<br>**4H(x)**:<br>Nb1-2 (2*d*): 1/3 2/3 3/4<br>0.5* Nb2-2 (4*f*): 1/3 2/3 0.502(2)<br>Se1-2 (4*e*): 0 0 0.570(1)<br>Se2-2 (4*f*): 2/3 1/3 0.681(1)	*R*p = 1.50 %;<br>*wR*p = 2.50 %;<br>For **4H(a)** ***R*1** = 8.35%;<br>for **4H(x)** ***R*1** = 10.45%	(1) The structure and the simulated powder XRD profile of the 4H(x) phase are shown in Supplementary figures 1,2. The CIF file of the mixed phase is available as Supplementary file 2.<br>(2) 4H(x) reflects disorder in stacking fault between 4H(a).<br>(3) Two phases essentially decrease both *R*p and *wR*p.	Present work

*Nb2 has 0.5 occupation

Supplementary figure 1

Representations of structures used for the Rietveld refinement.

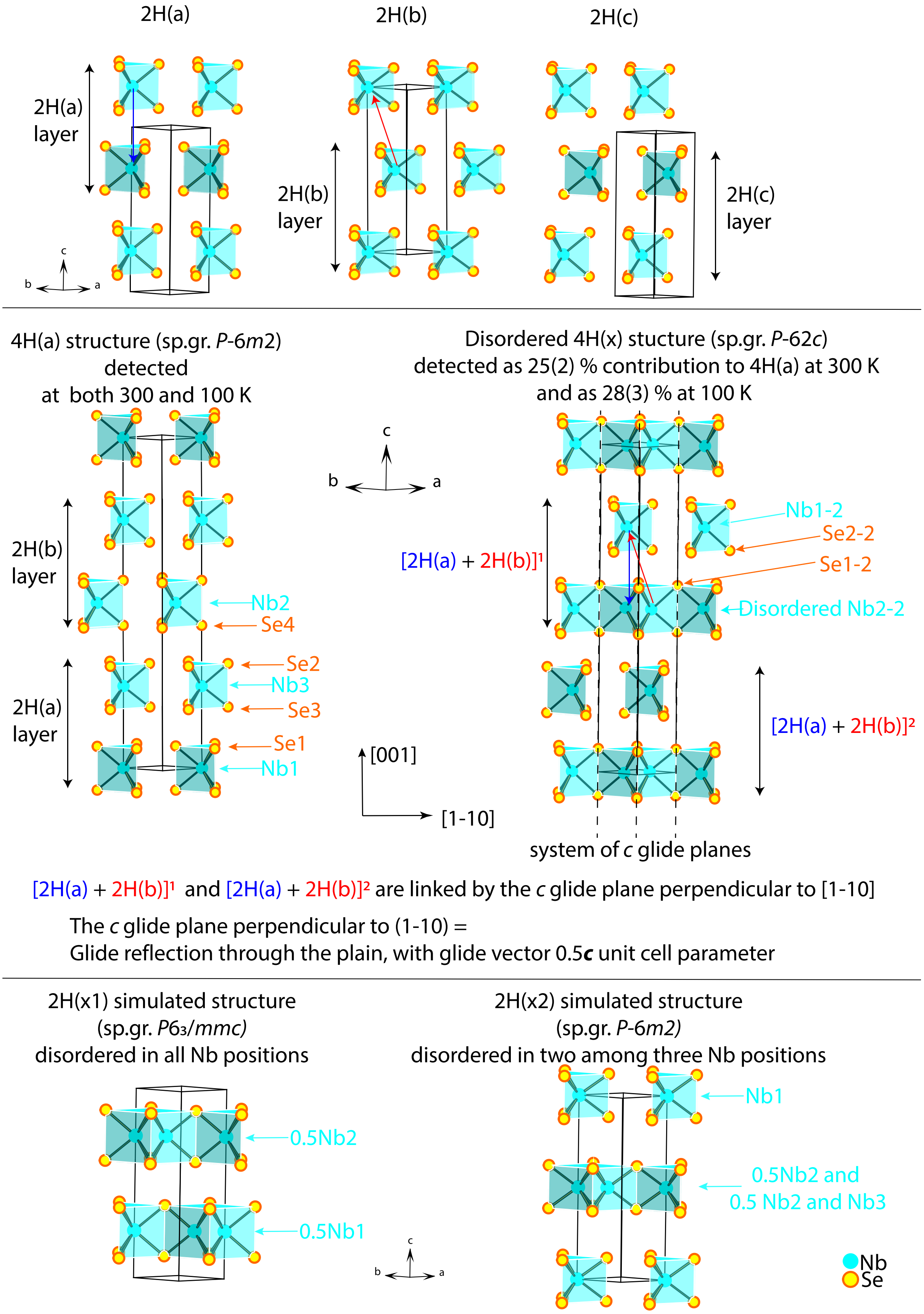

Supplementary figure 2

Simulated powder X-ray diffraction profiles of different structures used for the Rietveld refinement as well as the refined experimental diffraction profiles.

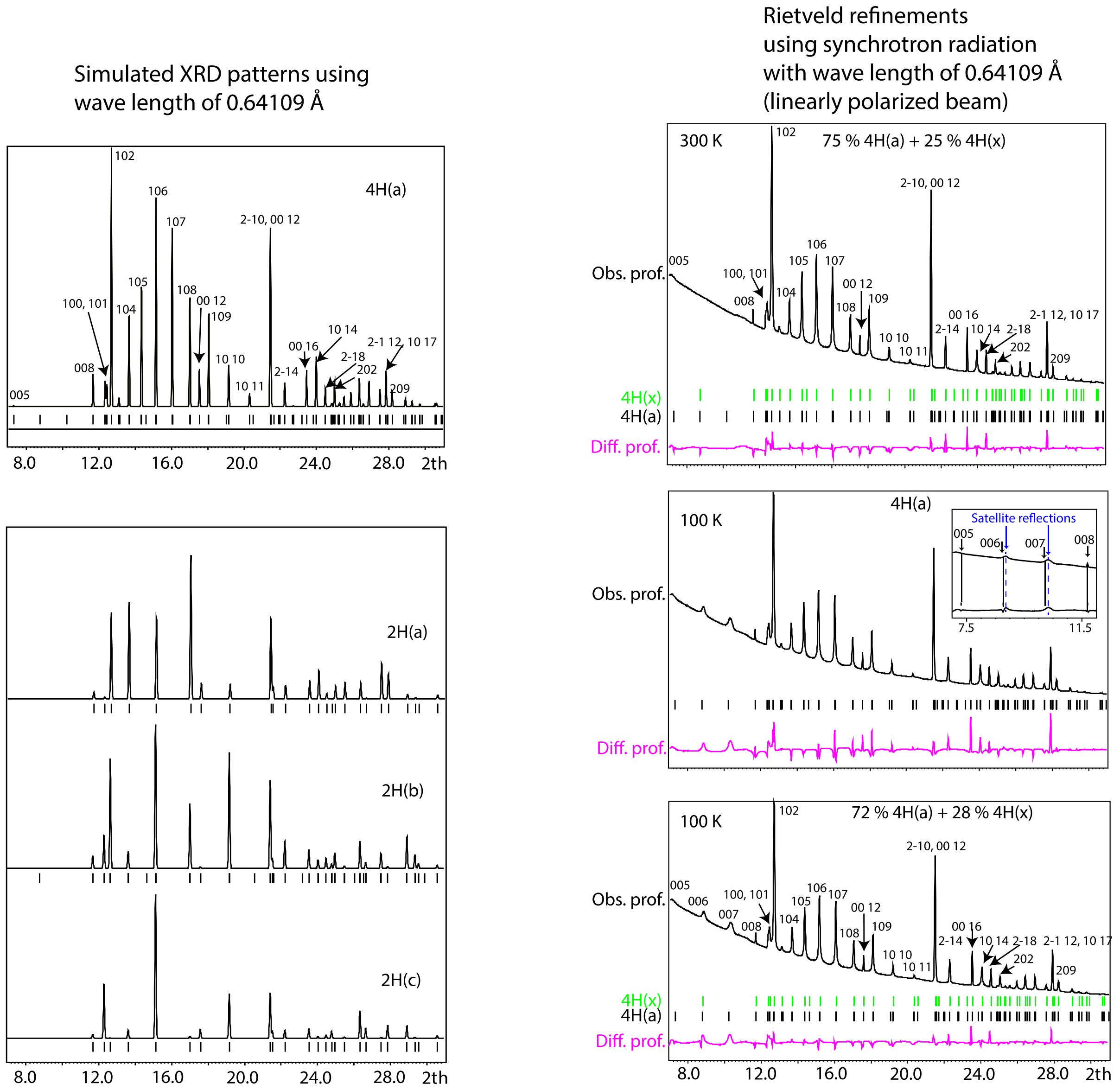

Supplementary references

1. Karn, N.K., Sharma, M.M., Felner, I., Awana, V.P.S. *J. Supercond. Novel Magn.* **37**, 1381-1391 (2024).

2. Zhou, M. et al. *J. Alloys Compd.* **991**, 174502 (2024).

3. Zhou, M. et al. Structures, charge density wave, and superconductivity of noncentrosymmetric 4H_a-$NbSe_2$. *Phys. Rev. B* **108**, 224518 (2023).
